# Exogenous ascorbic acid as a potent regulator of antioxidants, osmo-protectants, and lipid peroxidation in pea under salt stress

**DOI:** 10.1186/s12870-024-04947-3

**Published:** 2024-04-05

**Authors:** Rehana Kanwal, Muhammad Faisal Maqsood, Muhammad Shahbaz, Nargis Naz, Usman Zulfiqar, Muhammad Fraz Ali, Muhammad Jamil, Faizan Khalid, Qasim Ali, Muhammad Azeem Sabir, Talha Chaudhary, Hayssam M. Ali, Waleed A. A. Alsakkaf

**Affiliations:** 1 Department of Botany, The Islamia University of Bahawalpur, Bahawalpur, 63100 Pakistan; 2 Department of Botany, University of Agriculture, Faisalabad, 38040 Pakistan; 3 Department of Agronomy, Faculty of Agriculture and Environment, The Islamia University of Bahawalpur, Bahawalpur, 63100 Pakistan; 4 College of Agronomy, Northwest A&F University, Yangling, Xianyang, 712100 China; 5 Department of Soil Science, Faculty of Agriculture and Environment, The Islamia University of Bahawalpur, Bahawalpur, 63100 Pakistan; 6 Institute of Forest Sciences, The Islamia University of Bahawalpur, Bahawalpur, 63100 Pakistan; 7 Faculty of Agricultural and Environmental Sciences, Hungarian University of Agriculture and Life Sciences 2100, Godollo, Hungary; 8 Department of Botany and Microbiology, College of Science, King Saud University, Riyadh, 11451 Saudi Arabia

**Keywords:** Ascorbic acid, Salt stress, Photosynthetic pigments, Enzymatic antioxidants, Ionic homeostasis

## Abstract

Pea (*Pisum sativum* L.), a globally cultivated leguminous crop valued for its nutritional and economic significance, faces a critical challenge of soil salinity, which significantly hampers crop growth and production worldwide. A pot experiment was carried out in the Botanical Garden, The Islamia University of Bahawalpur to alleviate the negative impacts of sodium chloride (NaCl) on pea through foliar application of ascorbic acid (AsA). Two pea varieties Meteor (V1) and Sarsabz (V2) were tested against salinity, i.e. 0 mM NaCl (Control) and 100 mM NaCl. Three levels of ascorbic acid 0 (Control), 5 and 10 mM were applied through foliar spray. The experimental design was completely randomized (CRD) with three replicates. Salt stress resulted in the suppression of growth, photosynthetic activity, and yield attributes in pea plants. However, the application of AsA treatments effectively alleviated these inhibitory effects. Under stress conditions, the application of AsA treatment led to a substantial increase in chlorophyll *a* (41.1%), chl. *b* (56.1%), total chl. contents (44.6%) and carotenoids (58.4%). Under salt stress, there was an increase in Na^+^ accumulation, lipid peroxidation, and the generation of reactive oxygen species (ROS). However, the application of AsA increased the contents of proline (26.9%), endogenous AsA (23.1%), total soluble sugars (17.1%), total phenolics (29.7%), and enzymatic antioxidants i.e. SOD (22.3%), POD (34.1%) and CAT (39%) in both varieties under stress. Salinity reduced the yield attributes while foliarly applied AsA increased the pod length (38.7%), number of pods per plant (40%) and 100 seed weight (45.2%). To sum up, the application of AsA alleviated salt-induced damage in pea plants by enhancing photosynthetic pigments, both enzymatic and non-enzymatic activities, maintaining ion homeostasis, and reducing excessive ROS accumulation through the limitation of lipid peroxidation. Overall, V2 (Sarsabz) performed better as compared to the V1 (Meteor).

## Introduction

Soil salinity is a substantial obstacle that has a negative impact on the physiological and biochemical characteristics of plants, resulting in a decrease in yield. It also produces ionic imbalance, physiological drought, and osmotic stress, all of which interfere with essential plant cellular functions [1]. Increased concentrations of soluble salts, such as Na^+^, Ca^2+^, Mg^2+^, and Cl^−^, along with trace amounts of carbonates and nitrates, above a specified threshold (usually 0.15 g salt/100 g soil) in the topmost layer of soil are referred to as salinity [2]. The negative impacts of salinity in the soil are more noticeable in semi-arid or arid agricultural areas. When inadequate irrigation water is used, salt deposits accumulate in the soil, which hinders the plant’s ability to absorb water as well as essential nutrients [3].

It is estimated that salt-related issues already affect 20% of soils worldwide, and that figure will rise by 30% by the year 2050 [4]. Sea level rise and less precipitation are two main factors contributing to a global decline in productivity and these factors are linked to the intensifying consequences of salt stress [5]. In Pakistan, 6.68 million hectares of land are affected by salinity, and an additional 40,000 hectares of land degrade every year [6]. Salinity inhibits plant growth and creates barriers to sustainable use of land resources, resulting in a 1–2% annual rate of land degradation in Pakistan [5]. Salinity in the soil prevents plants from absorbing water, which lowers the water content of their cells and affects their turgor. Furthermore, salinity in the soil inhibits photosynthetic activity in plants and increases the production of reactive oxygen species (ROS), which further hampers the growth of plants [6].

Pea (*Pisum sativum* L.) is an important leguminous vegetable crop in the Fabaceae family and plays an important role in human diet. It has a long history of cultivation in various parts of the world, and it is mostly cultivated for its fresh and dry seeds [7]. Pea seeds are an excellent source of proteins, carbohydrates, dietary fiber, vitamins, and minerals. It is extremely susceptible to salt. Pea crop development and production have been significantly and negatively impacted by salt stress on a global scale [8]. Field peas reduce the risk of cardiovascular disease because it has low levels of salt, fat and cholesterol [9]. It is acknowledged as a restorer of the soil’s fertility due to its nitrogen fixation capacity through symbiotic relationships [10].

Pea being an important crop in Pakistan, has a considerable economic influence on agriculture. Punjab province is in first place with a 78.6% pea area and a 77.7% pea production. The main pea-growing regions in Punjab are Sheikupura, Nankana Sahib, Toba Tek Singh, Multan, Gujranwala, Sahiwal, Bahawalpur, Sialkot and Jhang. Peas are primarily grown in Punjab in the months of October and November [11]. The productivity of this essential crop is greatly impacted by a number of biotic and abiotic stress factors. Salinity is a particularly harmful abiotic stress because it severely limits crop development, growth and yield [12]. Pea yield has been observed to be reduced by 50% when exposed to moderate salt (100mM NaCl), but it is significantly decreased when exposed to high salinity [13]. Plants growing in saline soils undergo oxidative stress when salt builds up to dangerous levels within their cellular compartments [14]. This stress causes an increase in ROS, such as superoxide radicals, H_2_O_2_, singlet oxygen and OH^−^ radicals to toxic levels. These hamper normal plant growth by interfering with physiological and biochemical processes, including photosynthesis [15]. It primarily affects photosynthesis and CO_2_ diffusion by decreasing stomatal conductance, lowering net photosynthetic rate and disrupting photosystem II, thereby retarding these important processes [16]. Protein breakdown, membrane depolarization, lipid peroxidation, DNA mutation and other phytotoxic processes in plants are brought on by high ROS concentrations. When plants are subjected to such a constraint, they release antioxidants as a defence approach [17]. Important elements of this defence system that detoxify the harmful effects of ROS include proline, ascorbate peroxidase (APX), peroxidases (POD), superoxide dismutase (SOD) and catalase (CAT) [18]. Additionally, amino acids, proteins, and sugars also aid in efficiently detoxifying ROS induced by salinity [19]. Physiological drought is brought on by salt stress, which also lowers root water potential, causes phytotoxicity from excess Na^+^ and Cl^−^, disrupts nutrient uptake (K^+^, Ca^2+^, N and P) and hampers the vegetative and reproductive growth of plants [20, 21].

Plant growth regulators (PGRs) are small signaling molecules that profoundly affect the development and growth of plants. These effects include vascular patterning, growth characteristics, metabolic activities, flowering, fruit and seed development, and cell division and development [20]. Ascorbic acid (AsA) is widely acknowledged as the most effective growth regulator for mitigating the effects of salt stress [23]. Ascorbic acid activates a complex biological defence process and acts as an antioxidant [24]. It is used by many types of agricultural plants to mitigate the negative effects of salt stress [25]. Additionally, AsA serves as the first line of defence for plants to combat oxidative stress by removing many types of free radicals, primarily as a substrate of APX, a vital enzyme in the ascorbate glutathione pathway [26].

Legume plants are negatively affected by salt stress by interfering with the complex interactions between nutritional deficiencies, osmotic effects, hormones and specific ion toxicity [27]. Ascorbic acid is required for various activities that contribute to plant growth, including cell cycle control. Ascorbic acid treatment of soybean plants resulted in increased photosynthesis [28]. In fact, plants require ascorbate to survive, so AsA appears to be necessary for plant survival [29]. Exogenous AsA application on legume plants grown under salinity stress conditions increased salt tolerance, chlorophyll content, antioxidant enzyme activity and the ability to prevent abscisic acid build up and promoted the vegetative growth and productivity of different vegetable crops [30].

In plants, AsA has a variety of roles, especially in stressful situations. The current study aimed to address the significant gap in research regarding the foliar application of AsA to pea plants under salt stress. Concerning the significance of AsA, it is hypothesized that foliar application of ascorbic acid could mitigate the negative effects of salt stress and improve various physiological and biochemical attributes including pea plant growth, photosynthetic pigment content, antioxidant defence mechanisms, mineral nutrients uptake and ultimately yield potential under saline or non-saline conditions. To comprehensively investigate this hypothesis, various levels of AsA in pea plants with and without salt stress were examined. While previous studies have explored the impacts of exogenous AsA on salt tolerance in other crops, the specific effects on antioxidants, osmoprotectants, and lipid peroxidation in the currently studied verities have not yet been reported.

## Materials and methods

In order to assess the effects of salinity on pea plants through exogenous application of ascorbic acid (AsA), an experiment was carried out in the Botanical Garden, The Islamia University of Bahawalpur. A completely randomized design (CRD) with three replicates was used in this research. The study was completed from 22-Nov-2022 to 14-Feb-2023. Two varieties of pea i.e. Meteor (V1) and Sarsabz (V2) were treated with control and 100 mM NaCl stress. Three AsA concentrations (0, 5 and 10 mM) were applied through foliar spray. Plastic pots containing 8 kg of soil were utilized for sowing. Ten seeds in each pot were sown whereas seven plants were kept after two weeks of germination. After 45 days of sowing, salinity was maintained up to 100 mM in successive intervals in the form of solution. After two weeks of salinity application, a foliar spray of AsA in the form of a solution was applied. One plant from each replicate was carefully harvested for various growth attributes, i.e., chlorophyll pigments, biochemical traits, and antioxidant assessments after three weeks of AsA application, while yield attributes were collected when the crop reached maturity.

### Soil analysis

Soil analysis was performed at the Regional Agricultural Research Institute, Bahawalpur. For this purpose, soil used for current study was collected from Botanical Garden, The Islamia University of Bahawalpur. The soil’s pH was 7.98 and its electrical conductivity was 0.29 mS cm^− 1^. In addition to these physical attributes, other properties were also recorded such as organic matter (0.63%), available phosphorus (32 mg kg^− 1^), available potassium (28 mg kg^− 1^), saturated percentage (28%), and texture (sandy loam).

### Measurement of morphological traits

One plant from each pot was carefully uprooted and measuring tape was used to determine the lengths of plant’s shoot and root. The root and shoot fresh weights were calculated by means of an electrical balance. The dry weight of the shoot and root of plants was calculated after careful drying of samples at 65 °C in an oven (EYELA WFO-600ND).

### Determination of photosynthetic pigments

Carotenoids, chlorophyll *a*, chlorophyll *b* and the total chlorophyll were measured by using the Arnon’s [31] method. In 80% acetone, 0.1 g of leaf were ground up. The sample was kept overnight. Next day, reading was recorded by using spectrophotometer (IRMECO U2020, Germany) at 645, 663 and 480 nm.

### Evaluation of reactive oxygen species (H_2_O_2_, MDA)

Hydrogen peroxide (H_2_O_2_) was determined by using the Velikova et al. [32]. , technique. 0.25 g of fresh leaf sample was crushed in 2 ml of 0.1% TCA in ice. The supernatant was collected after 20 min of centrifuging at 1500 rpm. The test tube was filled with 1 mL of potassium iodide solution (165.9 g potassium iodide in 1 L of distilled water), 0.5 mL of phosphate buffer, and 0.5 mL of the leaf sample. Gently vortex before recoding the readings at 390 nm with a spectrophotometer (IRMECO U2020, Germany).

Malondialdehyde (MDA) composition was identified by Yagi [33]. A 0.25 g fresh leaf sample was crushed in 2 mL of 0.1% TCA (100 ml of distilled water and 0.1 g of TCA). After 20-minute centrifugation at 1500 rpm, remove the supernatant. A solution was made by mixing 20 g of TCA and 0.5 g of TBA with 100 ml of distilled water. In a test tube, add 4 mL of the solution and 1 mL of the centrifuged supernatant sample. For 30 min, incubate at 95 °C in a water bath. Then remove and allow to cool. Readings were taken at 532 nm and 600 nm through spectrophotometer (IRMECO U2020, Germany).

### Determination of enzymatic-antioxidants (SOD, POD, CAT)

Giannopolitis and Ries [34] technique was used to assess superoxide dismutase (SOD) activity. The reaction mixture was composed of 50 µL of nitroblue tetrazolium (NBT), 50 µL of riboflavin, 100 µL of L. methionine, 250 µL of phosphate buffer, 100 µL of Triton-X, 150 µL of distilled water, and 50 µL of the sample. The mixture was placed under light for 20 min and absorbance was recorded at 560 nm by using spectrophotometer (IRMECO U2020, Germany).

Chance and Maehly’s [35] method was used to measure the both peroxidase (POD) and catalase (CAT) activities. To determine POD activity, put 7.5 mL of phosphate buffer, 0.1 mL of guaicol solution (335 µL H_2_O_2_ + 15 mL phosphate buffer), 0.1 mL of H_2_O_2_ solution (100 mL H_2_O_2_ + 20 mL phosphate buffer), and 0.05 mL of sample extract in a cuvette. At 0, 30, 60, and 90 s intervals, absorbance was measured at 470 nm. For CAT determination, grind 0.2 g of the leaf sample in 5 mL of phosphate buffer. After 20 min of centrifuging at 1500 rpm, pour the supernatant into appendroffs. In a cuvette, add 1 mL of H_2_O_2_, 1.9 mL of phosphate buffer and 0.1 mL of the sample. At 240 nm, absorbance was measured at 0, 30, 60, and 90 s by using spectrophotometer (IRMECO U2020, Germany).

### Determination of ascorbic acid, total soluble proteins and sugars

According to Mukherjee and Choudhuri [36] protocol, AsA was measured. A 0.25 g fresh leaf sample was crushed in 5 mL of 6% trichloroacetic acid for extraction. In a test tube, 4 mL of the extract, 2 mL of 2% dinitrophenyl hydrazine in acidic medium and a drop of thiourea in 70% ethanol were added. The water bath was used for 15 min to heat the sample. The mixture was cooled to room temperature. The solution was store in ice at 0 °C and added 5 mL of 80% H_2_SO_4_ after cooling. At 530 nm, the absorbance value was recorded by using spectrophotometer (IRMECO U2020, Germany).

To measure total soluble proteins (TSP) bradford reagent was prepared by mixing 1 L of distilled water with 100 mL of 85% phosphoric acid, 0.1 g of Brilliant blue, and 50 mL of 95% ethanol. The freshly prepared reagent was filtered three to four times through filter paper. 0.25 g of leaf sample was crushed in 5 mL of phosphate buffer and then centrifuge to obtained supernatant. Each test tube was filled with 5 mL of reagent and 0.1 mL of supernatant and then vortex it. The absorbance at 595 nm was measured with a spectrophotometer (IRMECO U2020, Germany).

The method developed by Yemm and Wills [37] was used to calculate total soluble sugars (TSS). In a test tube, 0.5 g of leaves were boiled in 5 ml of distilled water for an hour. To make 25 mL, filter the extract and add water. Anthrone reagent (2.5 mL) and 0.5 mL of leaf extract were placed in each test tube. Again keep the test tube for 20–30 min in water bath. Cool and obtained readings at 620 nm by spectrophotometer (IRMECO U2020, Germany).

### Determination of total free proline and glycine betaine

To determine total free proline contents, fresh leaf material (0.25 g) was crushed and filtered after being dissolved in 5 mL of 3% sulfosalicylic acid. 1 mL of filtrate, 1 mL of acid ninhydrin, and 1 mL of glacial acetic acid were mixed in a test tube and heated in a water bath for 90 min at 100 °C. After that, 2 mL of toluene was added and samples were placed in ice to cool them. Two layers formed after the vortexing, and the spectrophotometer (IRMECO U2020, Germany) was used to measure the absorbance of the top pinkish layer at 520 nm.

To record the glycine betaine concentration, fresh material (0.25 g) was extracted in 5 ml of distilled water. At 12,000 rpm, the extract was centrifuged for 15 min. After mixing 2 ml of NH_2_SO_4_ with 1 ml of the sample, 500 µl of this extract was added to a test tube. Test tubes were cooled for 90 min following the addition of 0.2 ml of potassium tri-iodide. After that, test tubes were chilled on ice, add 2.8 ml of distilled water and 6 ml of 1, 2-dichloroethane. Two different layers were formed in a test tube, and the absorbance of lower layer was measured at 365 nm by using a spectrophotometer (IRMECO U2020, Germany).

### Determination of total phenolics, total flavonoids and anthocyanin

According to Julkenen-Titto [38], the amount of total phenolic was recorded. A 10 mL solution of 80% acetone was used to extract the leaf material of 0.5 g. The supernatant (1 mL) was mixed with 5 mL of Na_2_Co_3_ (20%), 1 mL of Folin-Ciocalteu phenol reagent, and 1 mL of the supernatants. To make the mixture 10 mL in volume, distilled water was utilized. The absorbance of the reaction mixture was measured at 750 nm.

To measure flavonoids, 300 µL of NaNO_3_ and 1 ml of the ethanol extract was added in a test tube, followed by a short incubation at 25 °C. Following the addition of 300 µL of AlCl_3_, for 5 min, the mixture was maintained at room temperature. The mixture has been left to cool at room temperature for 10 min after adding 2 ml of NaOH (1 M). The mixture was diluted to a level of 10 mL using distilled water. At 510 nm, the absorbance was measured with the help of spectrophotometer (IRMECO U2020, Germany) [39].

Anthocyanin contents were recorded according to Murray and Hackett [40]. In 5 ml of acidified methanol, 0.2 g of the leaf sample was crushed. By dissolving 1 mL of HCL in 120 mL of methanol, acidified methanol was prepared. After labelling the test tube carefully, transfer the sample into it. Then, test tube was placed in water bath at 50 °C for 60 min. The spectrophotometer (IRMECO U2020, Germany) was used to record absorbance at 535 nm.

### Ion analysis of root and shoot (Na^+^, Ca^2+^, K^+^)

In separate labeled conical flasks, 0.1 g of oven-dried root and shoot samples were mixed in 5 ml of pure H_2_SO_4_ and leave the flask covered overnight. Next day, place the flasks on a hot plate at 100 °C and gradually add pure H_2_O_2_ to make the samples transparent. After cooling filter the solution by using filter paper and add distilled water to maintain the volume up to 50 ml. The Na^+^, Ca^2+^, and K^+^ ions in the root and shoot samples were measured by using a flame photometer (Sherwood-410, UK).

### Estimation of yield-related attributes

The number of pods per plant were counted, and measuring tape was used to determine the length of each pod from the base to the tip. 100 seeds for each treatment were counted and weighed by using an electrical balance.

### Statistical analysis

A completely randomized design (CRD) with three replicates was used in the experiment. Using the USA Statistix software (version 8.1), a three-way analysis of variance (ANOVA) was used to analyze the data. A significance level of 5% was used to compare the mean treatments. Data were presented graphically using Microsoft Excel (Version 2019). The statistical program ‘Origin 2023’ was used for correlation matrix and radar analysis.

## Results

### Morphological parameters

**Fig. 1:**
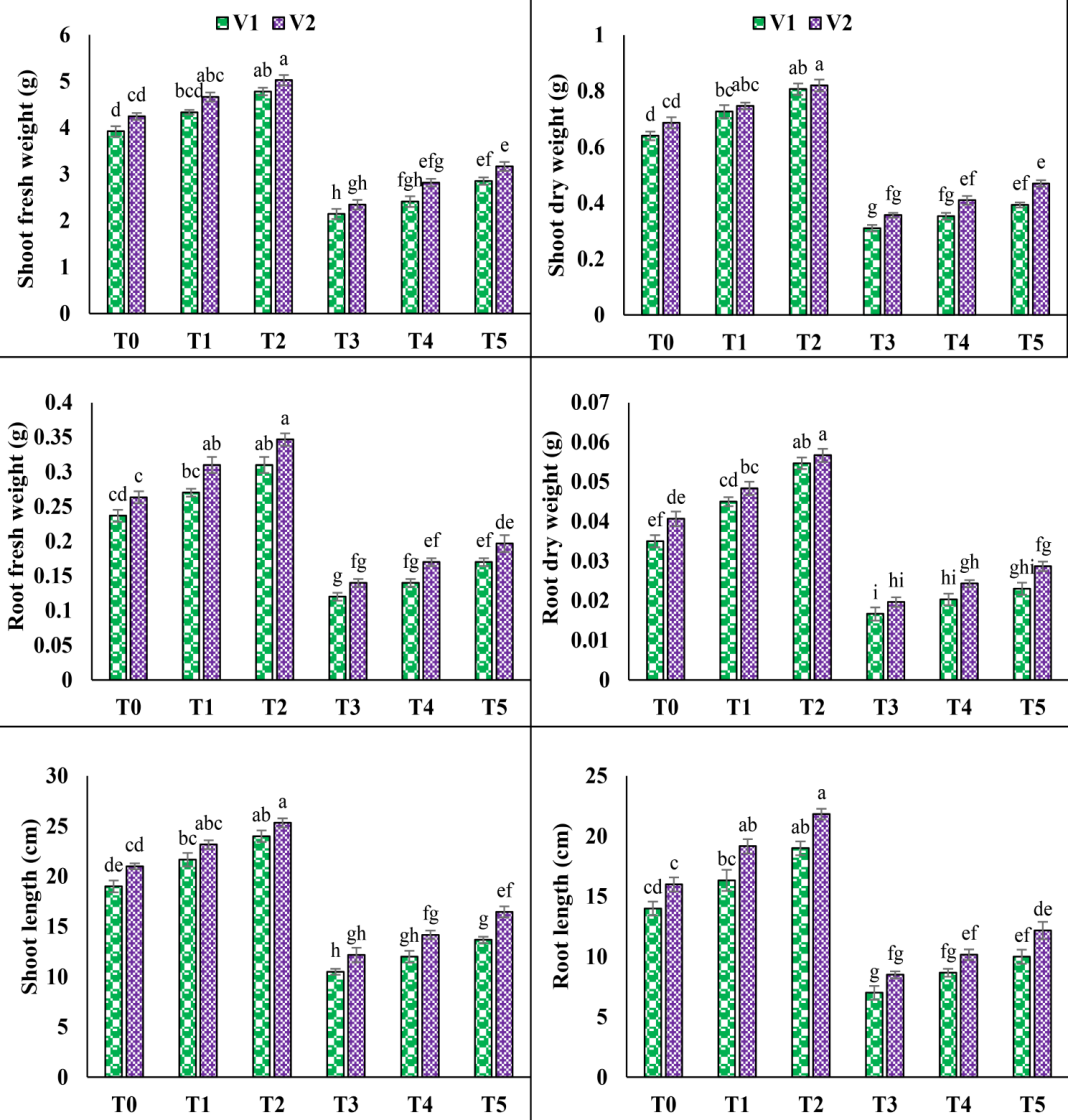
Effect of ascorbic acid (AsA) on shoot fresh weight, shoot dry weight, root fresh weight, root dry weight, shoot length and root length of pea under salt stress. The three replicates ± SE is shown by the error bars above the means. For a parameter, means that share same letters do not differ significantly at *p* < 0.05. (V1 = Meteor; V2 = Sarsabz; T0 = Control + 0 mM AsA, T1 = Control + 5 mM AsA, T2 = Control + 10 mM AsA, T3 = 100 mM NaCl + 0 mM AsA, T4 = 100 mM NaCl + 5 mM AsA and T5 = 100 mM NaCl + 10 mM AsA)

The application of 100 mM NaCl (T3) showed considerable reduction in the root and shoot lengths, root and shoot fresh and dry weights in both varieties. A significant decrease observed in root length, root fresh and dry weight (50%, 49.2% and 52.3%) in V1 and 46.8%, 46.8% and 51.6% in V2, respectively. It is also noticed that salinity reduced the shoot length, shoot fresh and dry weights in V1 (44.7%, 45.3% and 51.5%) and V2 (42%, 44.7% and 48%). However, foliar application of AsA (T5) increased the root length, root fresh and dry weights in V1 up to 42.8%, 41.6% and 38%, and 43.1%, 40.4% and 45.7%, respectively in V2. Likewise, under T5 foliar application of AsA also improved the shoot length, shoot fresh and dry weights up to 30.1%, 33.1% and 26.8% in V1 and 35.3%, 34.9% and 31.7% in V2, accordingly (Fig. 1).

### Photosynthetic pigments and ROS

**Fig. 2:**
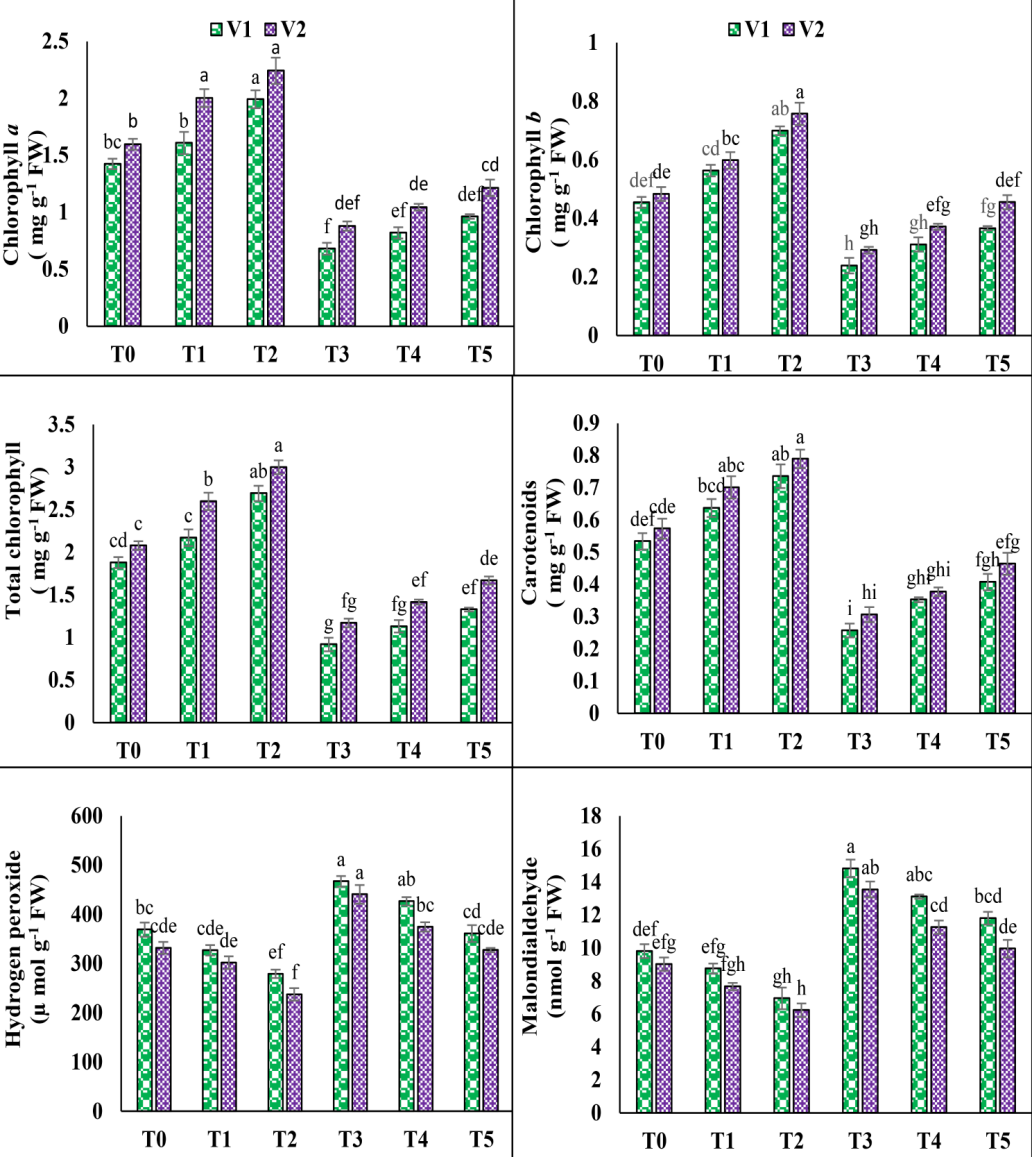
Effect of ascorbic acid (AsA) on chlorophyll *a*, chlorophyll *b*, total chlorophyll, carotenoids, hydrogen peroxide and malondialdehyde of pea under salt stress. The three replicates ± SE is shown by the error bars above the means. For a parameter, means that share same letters do not differ significantly at *p* < 0.05. (V1 = Meteor; V2 = Sarsabz; T0 = Control + 0 mM AsA, T1 = Control + 5 mM AsA, T2 = Control + 10 mM AsA, T3 = 100 mM NaCl + 0 mM AsA, T4 = 100 mM NaCl + 5 mM AsA and T5 = 100 mM NaCl + 10 mM AsA)

Results showed that chlorophyll *a* and *b*, total chlorophyll, and carotenoids levels were significantly reduced when both pea varieties were subjected to 100 mM salt stress (T3). Under salinity, the content of chlorophyll *a* and *b*, total chlorophyll, and carotenoids decreased by up to 52.2%, 47.5%, 51.1%, and 51.8% in V1, and 44.9%, 39.6%, 43.7%, and 46.5% in V2, accordingly. However, under T5, the exogenous application of 10 mM AsA resulted in improvements in the contents of chlorophyll *a* and *b*, total chlorophyll, and carotenoids in V1, reaching levels of 41.5%, 53.5%, 44.6%, and 58.4%, and in V2, with values of 38.2%, 56.1%, 42.7%, and 51.4%, respectively. Conversely, salinity stress (T3) resulted in elevated oxidative damage, as evidenced by increased levels of reactive oxygen species (H_2_O_2_ and MDA) in both varieties. The levels of H_2_O_2_ and MDA exhibited a notable increase of 26.5% and 51.2% in V1, and 32.7% and 50.2% in V2, correspondingly, in response to exposure to 100 mM NaCl stress. However, under T5, the foliar application of 10 mM AsA alleviated the adverse impact of salinity, resulting in a reduction of H_2_O_2_ and MDA contents by 22.6% and 20.3% in V1, and 25.6% and 26.4% in V2, respectively (Fig. 2).

### SOD, POD, CAT, TSP, TSS and flavonoids

**Fig. 3:**
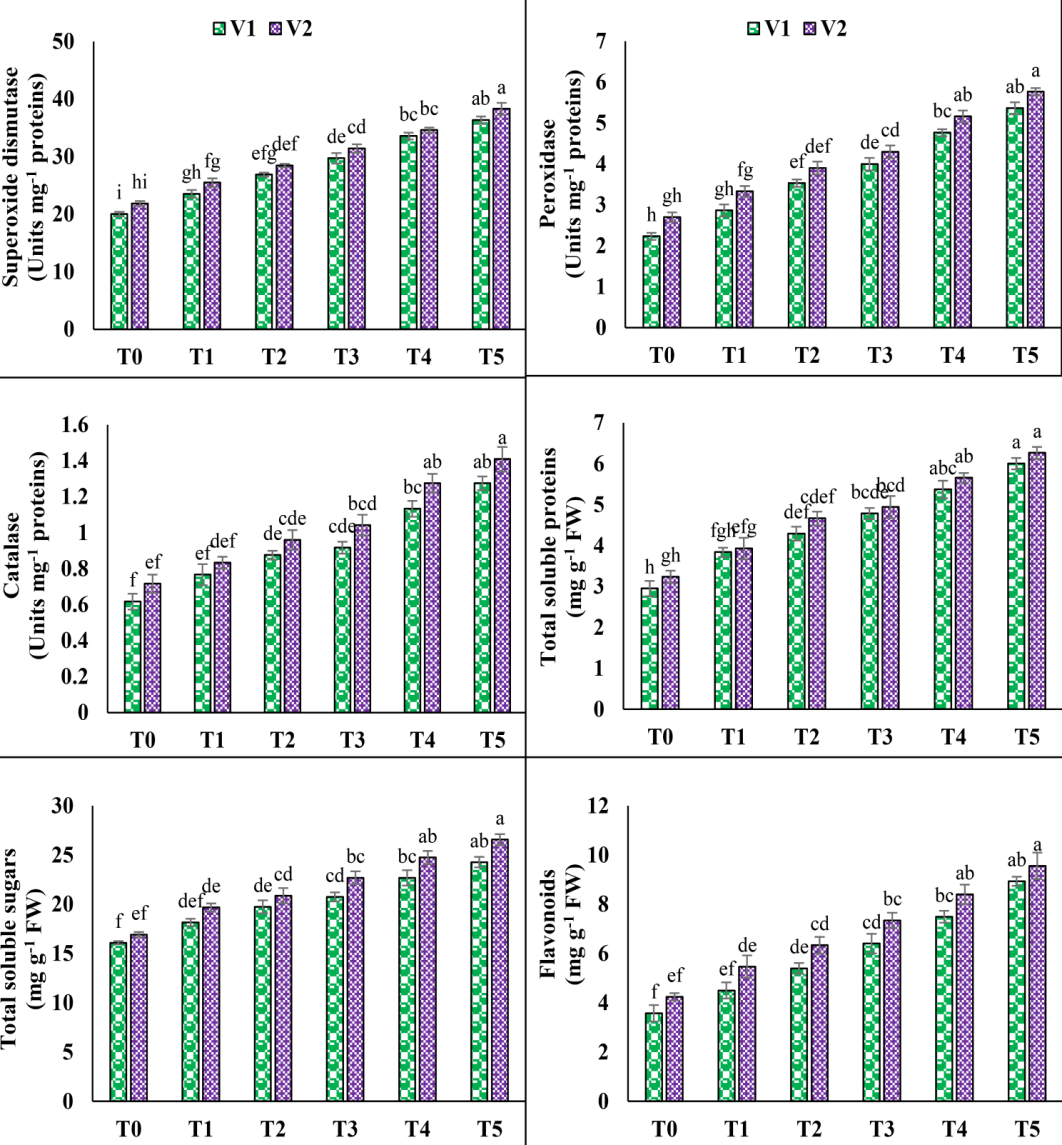
Effect of ascorbic acid (AsA) on superoxide dismutase (SOD), peroxidase (POD), catalase (CAT), total soluble proteins (TSP), total soluble sugars (TSS) and flavonoids of pea under salt stress. The three replicates ± SE is shown by the error bars above the means. For a parameter, means that share same letters do not differ significantly at *p* < 0.05. (V1 = Meteor; V2 = Sarsabz; T0 = Control + 0 mM AsA, T1 = Control + 5 mM AsA, T2 = Control + 10 mM AsA, T3 = 100 mM NaCl + 0 mM AsA, T4 = 100 mM NaCl + 5 mM AsA and T5 = 100 mM NaCl + 10 mM AsA)

In both pea varieties, the application of salinity (T3) markedly boosted the activities of SOD, POD and CAT. V1 showed 48.4%, 79.1% and 48.6% increase in SOD, POD and CAT activities under T3 while V2 experienced 43.7%, 59.2% and 45.3% increase in SOD, POD and CAT activities, accordingly. The activities of SOD, POD, and CAT were considerably elevated by the application of 10 mM ascorbic acid by 22.3%, 34.1%, and 39% in V1 and 21.9%, 34.1%, and 35.2% in V2 under T5, correspondingly. Both pea plant varieties showed a significant increase in TSP, TSS, and flavonoids under 100 mM salt stress. Under T3, V1 increased TSP, TSS, and flavonoids by 62.1%, 28.9%, and 79.3%, respectively. Meanwhile, V2 increased TSP, TSS, and flavonoids by 52.7%, 34.1%, and 73.3%, accordingly. Foliar treatment of 10 mM ascorbic acid enhanced TSP, TSS, and flavonoids by 25.5%, 17%, and 39.4% in V1 and 26.8%, 17.1%, and 30.1% in V2, respectively, under T5 (Fig. 3).

### GB, proline, anthocyanin, ascorbic acid and phenolics

**Fig. 4:**
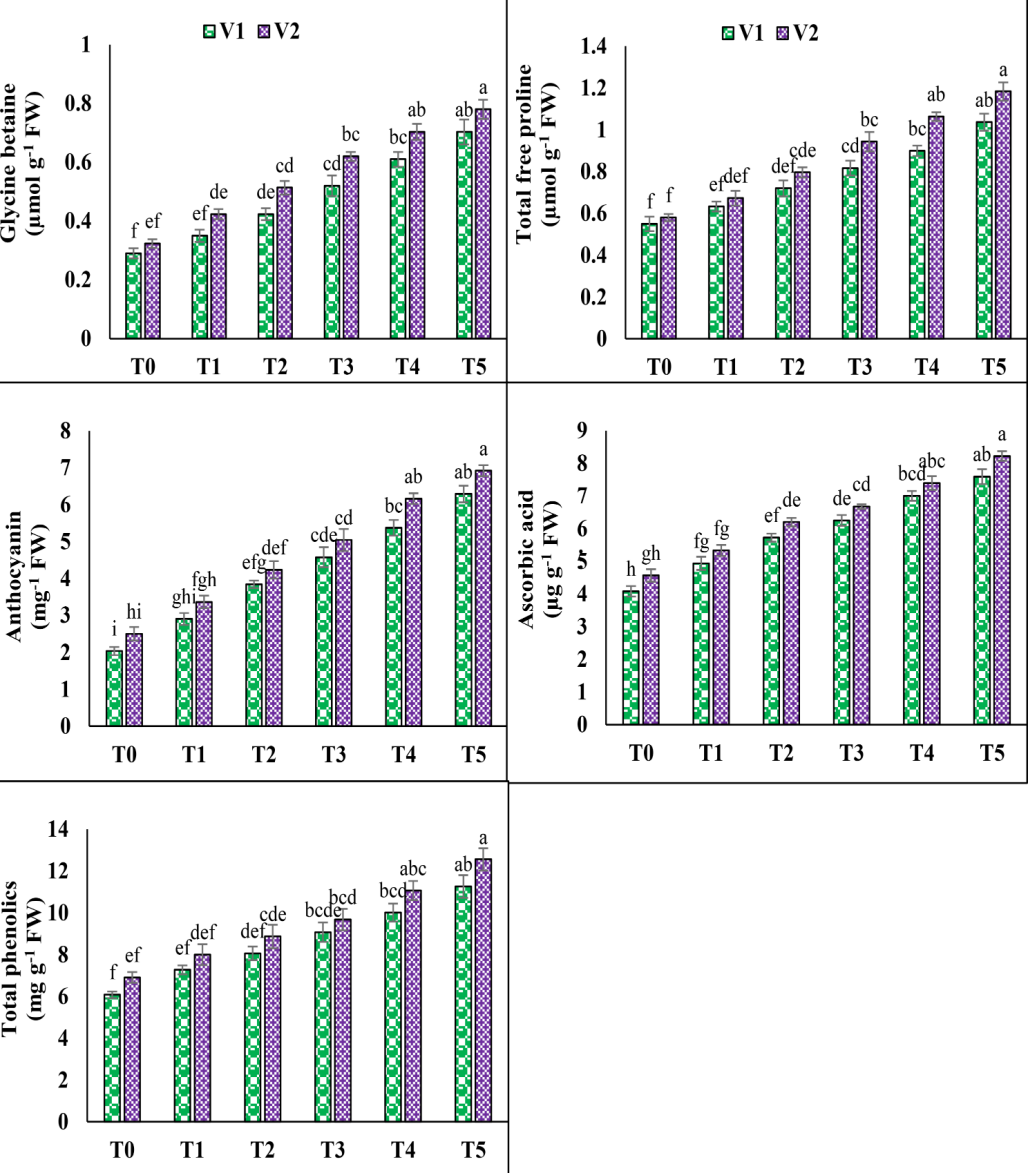
Effect of ascorbic acid (AsA) on glycine betaine, total free proline, anthocyanin, ascorbic acid and total phenolics of pea under salt stress. The three replicates ± SE is shown by the error bars above the means. For a parameter, means that share same letters do not differ significantly at *p* < 0.05. (V1 = Meteor; V2 = Sarsabz; T0 = Control + 0 mM AsA, T1 = Control + 5 mM AsA, T2 = Control + 10 mM AsA, T3 = 100 mM NaCl + 0 mM AsA, T4 = 100 mM NaCl + 5 mM AsA and T5 = 100 mM NaCl + 10 mM AsA)

Both varieties of pea plants increased their glycine betaine, total free proline, and anthocyanin levels after being exposed to 100 mM salt (T3). Statistical data showed that under T3, V1 increased its contents of glycine betaine, total free proline, and anthocyanin by up to 79.3%, 48.4%, and 124.5%, whereas V2 increased its concentrations by up to 91.7%, 48.6%, and 101.5%, accordingly. Under saline circumstances (T5), the application of 10 mM AsA significantly increased the glycine betaine, total free proline, and anthocyanin contents in V1 (35.2%, 26.9%, and 37.4%) and V2 (25.8%, 25.4%, and 37.2%). Likewise, under T3, V1 showed an increase of 53.1% in ascorbic acid and 49% in phenolics, while V2 exhibited increases of 46.1% in ascorbic acid and 40.2% in phenolics. However, the exogenous application of AsA under T5 resulted in a notable increase in both ascorbic acid and phenolics contents, reaching up to 21.4% and 24.1% in V1, and 23.1% and 29.7% in V2, correspondingly (Fig. 4).

### Root and shoot ions (Na^+^, K^+^ and Ca^2+^)

**Fig. 5:**
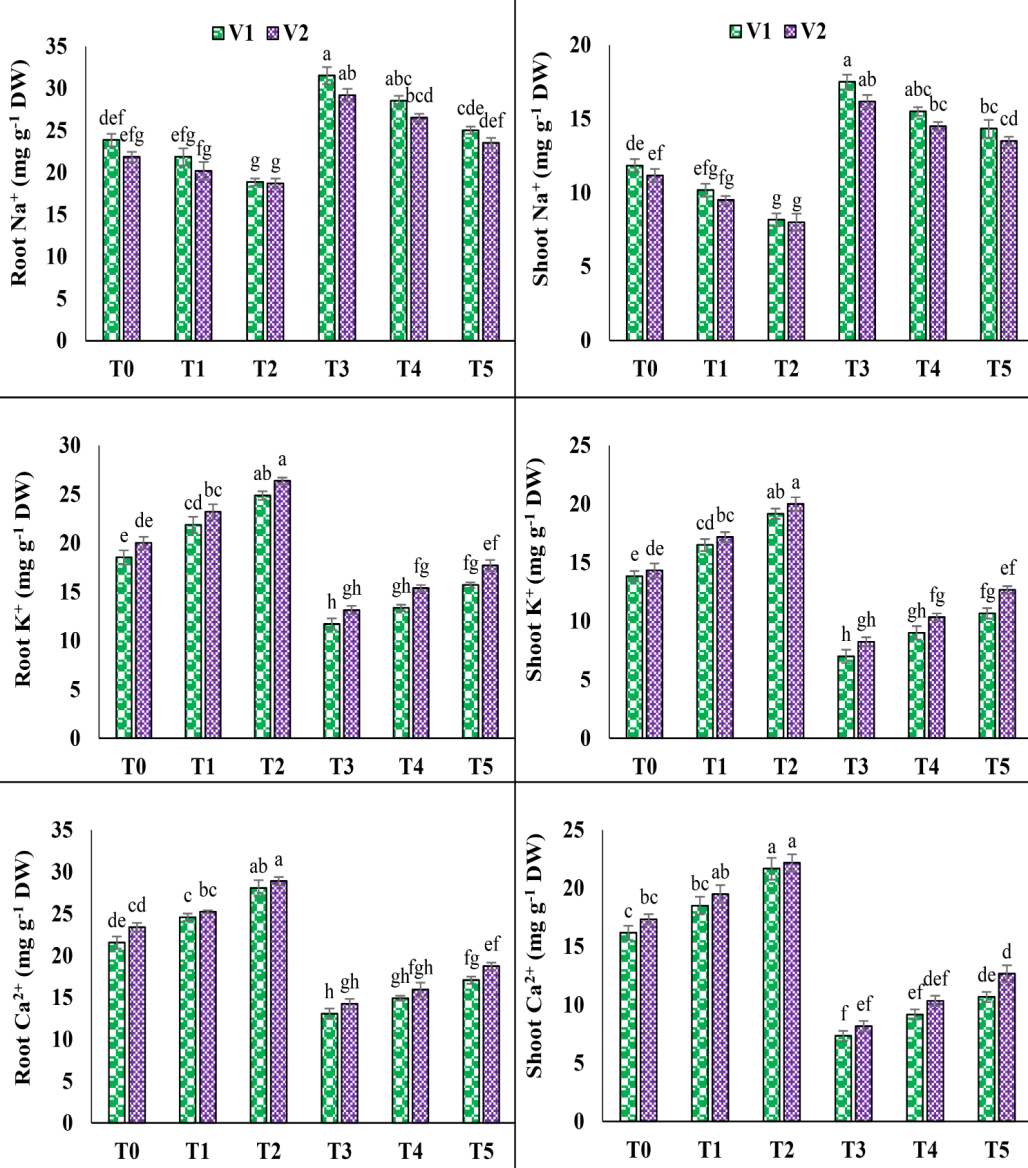
Effect of ascorbic acid (AsA) on root sodium, shoot sodium, root potassium, shoot potassium, root calcium and shoot calcium of pea under salt stress. The three replicates ± SE is shown by the error bars above the means. For a parameter, means that share same letters do not differ significantly at *p* < 0.05. (V1 = Meteor; V2 = Sarsabz; T0 = Control + 0 mM AsA, T1 = Control + 5 mM AsA, T2 = Control + 10 mM AsA, T3 = 100 mM NaCl + 0 mM AsA, T4 = 100 mM NaCl + 5 mM AsA and T5 = 100 mM NaCl + 10 mM AsA)

The application of 100 mM NaCl under T3 resulted in a substantial increase in harmful Na^+^ in the root and shoot of both varieties. A significant increase observed in root and shoot Na^+^ (32.1% and 47.8%) in V1 and 33.5% and 44.7% in V2, respectively. In V1, however, the contents of root and shoot Na^+^ were declined up to 20.6% and 18%, and 19.4% and 16.4%, respectively, in V2, with the help of foliar application of AsA under T5. It is also noticed that salinity reduced the beneficial root and shoot K^+^, as well as root and shoot calcium Ca^2+^ in V1 (36.8%, 49.3%, 39.4% and 54.6%) and V2 (34.4%, 42.5%, 39.1% and 52.8%) accordingly. In contrast, under T5 foliar application of AsA improved the root and shoot K^+^, root and shoot Ca^2+^ up to 34.1%, 52.3%, 30.6% and 45.4% in V1 and 34.7%, 53.8%, 31.6% and 55.1% in V2, respectively (Fig. 5).

### Yield parameters

**Fig. 6:**
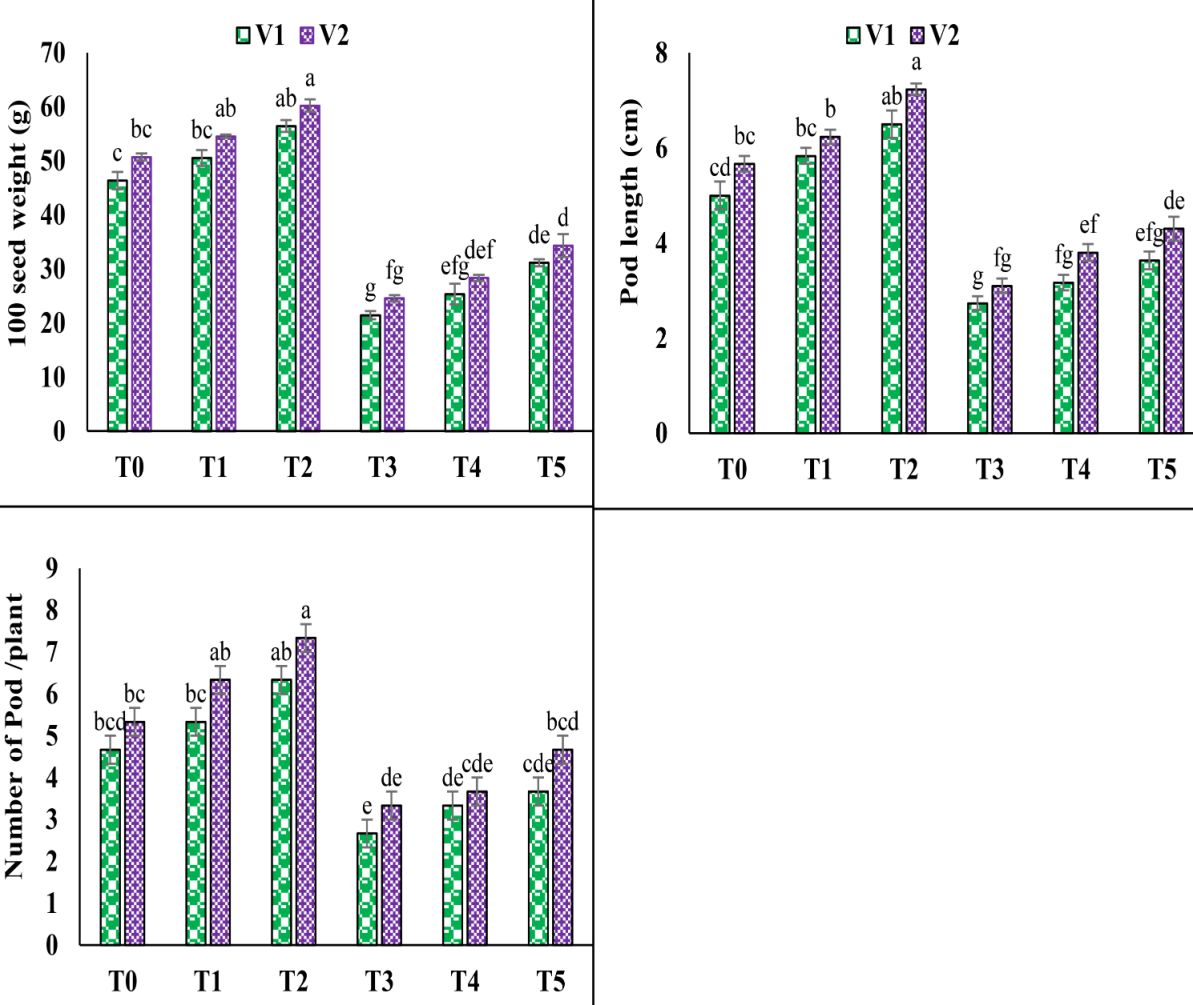
Effect of ascorbic acid (AsA) on hundred seed weight, pod length and number of pods of pea under salt stress. The three replicates ± SE is shown by the error bars above the means. For a parameter, means that share same letters do not differ significantly at *p* < 0.05. (V1 = Meteor; V2 = Sarsabz; T0 = Control + 0 mM AsA, T1 = Control + 5 mM AsA, T2 = Control + 10 mM AsA, T3 = 100 mM NaCl + 0 mM AsA, T4 = 100 mM NaCl + 5 mM AsA and T5 = 100 mM NaCl + 10 mM AsA)

The overall yield of pea plants was adversely affected by the 100 mM NaCl stress. According to the statistics, salt stress significantly reduced the 100 seed weight, pod length, and number of pods per plant under T3. A substantial decline in the 100 seed weight, pod length, and number of pods per plant under salinity stress was seen in V1 (53.7%, 45.3%, and 42.8%) and V2 (51.5%, 45.2%, and 37.5%), correspondingly. The foliar application of 10 mM of AsA under T5 showed the significant increase in the 100 seed weight, pod length and number of pods per plant up to 45.2%, 32.9% and 37.5% in V1 and V2 up to 39.6%, 38.7% and 40%, accordingly (Fig. 6).

**Fig. 7:**
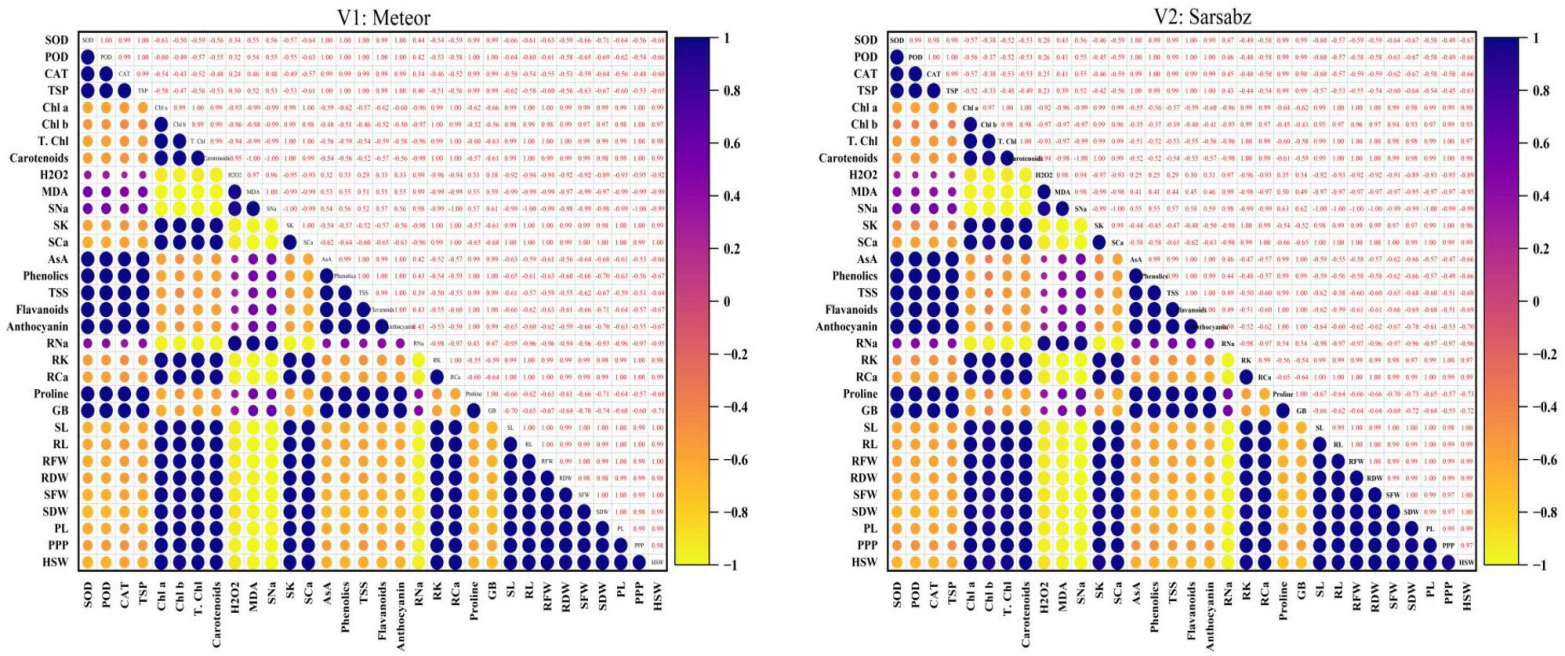
Correlation matrix of various parameters of pea varieties (**A**) Meteor (V1) (**B**) Sarsabz (V2) and different levels of ascorbic acid (AsA) under salt stress conditions

The correlation analysis revealed that the two varieties of pea studied traits had a similar correlation trend. Pearson’s correlation of antioxidants, non-enzymatic and biochemical traits with plant morphological and yield parameters was analyzed for the two varieties of pea (Fig. 7). In pea plants, a highly positive correlation was observed between photosynthetic pigments (chlorophyll *a*, *b*, total chlorophyll, and carotenoids) with morphological parameters (SL, SFW, SDW, RL, RFW and RDW) and yield increasing these attributes directly correlated with the yield plant biomass and increased significantly (Fig. 7). A strong negative correlation was found between ROS (H_2_O_2_, MDA), enzymatic antioxidants (CAT, SOD, POD), non-enzymatic antioxidants (AsA, Anthocyanin, TSS, TSP, Proline, GB, Phenolics, Flavonoids), with morphological parameters of pea. Salt ions shows variation in correlation as Ca^2+^ and K^+^ ions for root and shoot showed a positive correlation while the Na^+^ ion for root and shoot showed a negative correlation with morphological parameters of plants. The enzymatic antioxidants showed a significant strong correlation with non-enzymatic antioxidants (Fig. 7). Under salinity an increase in enzymatic and non-enzymatic antioxidant will cause a decline in photosynthetic pigments (chlorophyll *a*, *b*, total chlorophyll, and carotenoids) which will directly cause a decline in morphological parameters and decline in yield. The SNa, MDA, and H_2_O_2_ showed a strong negative correlation with all plant biomass attributes.

**Fig. 8:**
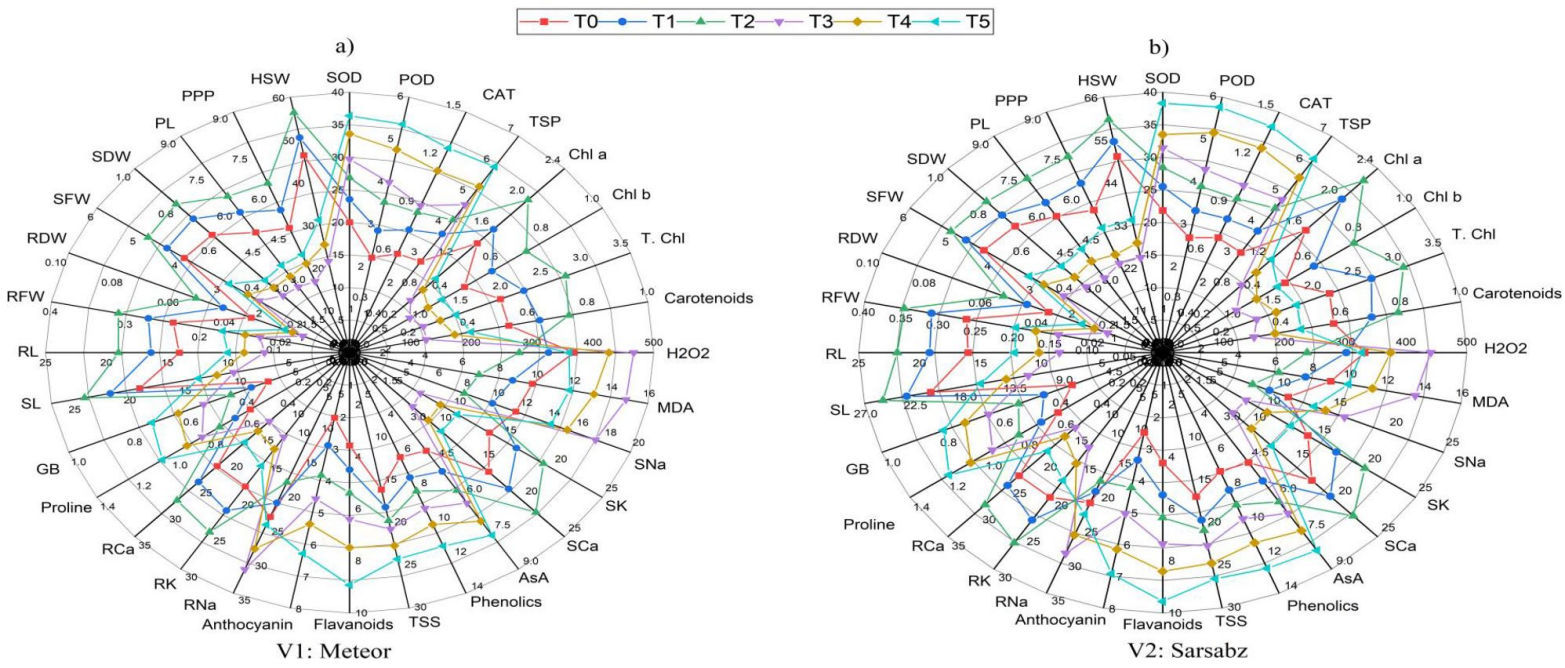
Radar chart showing the impact of salinity and ascorbic acid treatments on various parameters of pea varieties (**A**) Meteor (**B**) Sarsabz

The radar analysis presented the average observations of all parameters studied under foliar application of AsA to enhance salinity tolerance in peas which including morphological traits, photosynthetic pigments, ROS including H_2_O_2_ and MDA, enzymatic antioxidants, non-enzymatic antioxidants, salt ions and yield related traits (Fig. 8). According to the findings, the morphological traits such as root/shoot length, root/shoot fresh and dry weight of the pea plant increased under treatment T2, followed by T1 and control for both the varieties. The photosynthetic pigments also followed the same increasing trend for the same. The treatments T4 and T5 had decreased the photosynthetic pigments, while simultaneously increasing the activity of both enzymatic and non-enzymatic antioxidants such as SOD, POD, CAT, TSP, TSS, Total phenolics, anthocyanin and flavonoids as shown in (Fig. 8). The plant ions (Na^+^) for root and shoot has been increased under treatment T3, T4 and T5 while (Ca^2+^ and K^+^) have been increased under T1, T2 and control conditions. The contents for proline and GB also increased under treatment T4 and T5 while reduced in control, T1 and T2.

## Discussion

The increasing application of fertilizers to improve crop yield has caused a rise in the amount of soluble salt in groundwater and soil, which eventually enhance the salinity level of the soil [2]. Crop growth, physiological development and yield are all impaired by the increasing soil salinity [1]. Soil salinity decreased the morphological traits i.e., shoot and root length, shoot and root fresh weight and shoot and root dry weight of both pea varieties. This reduction is due to the combined results of osmotic and ionic stress caused by limited water and nutrient intake [41], as well as lower turgor pressure [42], reduced photosynthetic activity, Na^+^ ion accumulation [43] and disintegration of cell membranes under salinity [44]. Foliar application of AsA lowered the hostile effects of salinity and enhanced the morphological parameters of both pea varieties under both control and saline conditions.

Both varieties of pea showed decreased amount of photosynthetic pigments i.e., total chlorophyll, chlorophyll *a*, chlorophyll *b* and carotenoids. Decreased amount of photosynthetic pigments can be associated to the thylakoid membrane being damaged by excessive salinity [13], which results in decreased CO_2_ absorption [16], immediate stomatal closure and the deactivation of enzymes necessary for dark-reaction [45]. Moreover, under stress and control environment, exogenous application of AsA increased the amount of total chlorophyll and carotenoid contents. Exogenous AsA consistently regulates stomatal opening, reduces transpiration, keeps turgor intact, and eventually enhances plant growth and productivity under stress conditions [46]. It also shields the photosynthetic pigment machinery as it serves as a substrate for the enzyme APX, which scavenges the ROS that generates in the thylakoid membranes [47].

Under salt stress, both varieties of pea had higher levels of reactive oxygen species (H_2_O_2_ and MDA) that indicated the oxidative stress. Osmotic stress, ion toxicity and nutritional imbalance all impair the structural integrity of a number of biomolecules [13]. Furthermore, these variables disturb the metabolic, biochemical and physiological functions of cellular components, which results in an excess of ROS formation and additional substantial damage to the components of the cell [48, 49]. Foliar AsA spray reduced the concentration of H_2_O_2_ and MDA, and ultimately decreased the oxidative stress and increased the growth and development of pea plants under both control and salt stress. Ascorbic acid treatment of salt-treated plants decreased the levels of MDA and H_2_O_2_ as it improved the antioxidant defence system by scavenging ROS and neutralizing the lipid peroxy radicals [50]. It directly protects membranes from lipid peroxidation and played a substantial role in lowering oxidative stress [51].

Under salt stress, both pea varieties have higher activities of enzymatic antioxidants including SOD, POD and CAT. These enzymatic antioxidants are crucial in protecting the plants from damaging impacts of ROS [52]. The enzyme SOD removes the superoxide ions by converting them into the less harmful products like oxygen and H_2_O_2_ [53]. Both POD and CAT detoxify hydrogen peroxide (H_2_O_2_), generated in the dismutation process through SOD by turning it into water and oxygen, therefore playing an essential role in ROS scavenging under salt stress [54]. Exogenously applied AsA improved the activity of SOD, POD and CAT in both varieties of *+* under control and salinity. The stability between ROS production and elimination is necessary for plants to endure and thrive in hostile environments [55]. Stress tolerance is connected to AsA accumulation because AsA scavenges ROS and increases oxidative defence capability, hence promoting plant growth and development under stress conditions [56].

It was observed that the levels of endogenous ascorbic acid (AsA), TSP, and TSS improved under salt stress. Endogenous oxidative protection by AsA is a crucial factor in determining a plant’s viability during stress. The AsA rapidly decrease the ROS and protects biomolecules from being harmed prior to the activation of antioxidant enzymes [57]. Total soluble protein (TSP) accumulation in the presence of salinity can be regarded as a type of nitrogen storage that is beneficial when plant has to face stress and may contribute to osmotic adjustment [2, 58]. Under salt stress, plants may produce more TSS, which is certainly an osmo-protective mechanism [19] compensating an elevated salt intake [59]. Foliar treatment of AsA under both control and salt stress enhanced the level of endogenous ascorbic acid (AsA), TSP, and TSS in pea plants. Foliar application of AsA decreases stress and increases the production of endogenous AsA within plant cells. Ascorbic acid shields plant organelles and cells from ROS that accumulates excessively as a result of oxidative stress, thus helping to survive with stressful circumstances [60]. Ascorbic acid influences several enzymes [61], stimulates a variety of physiological and signaling pathways [62], increases the overall amount of proteins and carbohydrates [63] and works synergistically with other antioxidants to reduce oxidative stress [64].

Salt stress led to a significant rise in total free proline and glycine betaine. The increasing levels of proline and glycine betaine under salinity reduced the detrimental effects of salt stress [65], supporting the idea that proline and GB play a significant role in controlling plant growth and maintain cell turgor [66]. Increased antioxidant defence system, reduced oxidative stress, enhanced synthesis of suitable solutes, and accelerated proline accumulation, all are possible strategies to counteract the reduction in plant growth induced by salinity [67]. The application of AsA enhanced the total free proline and glycine betaine under both saline and non-saline environment. Ascorbic acid application on pea leaves, increased the various osmolytes, including glycine betaine and proline [68] and this is associated to plant tolerance improvement by contributing to cellular osmotic adjustment [69], ROS detoxification [70], membrane integrity protection [71], nutritional homeostasis [72], ion compartmentalization [15], and enzyme/protein stabilization.

Total phenolics, flavonoids, and anthocyanin levels increased significantly in both varieties of pea under salt stress. These are important secondary compatible plant metabolites that shield plants from abiotic challenges and prevent the generation of ROS [73] and possibly prevent the lipid peroxidation in stressed plants and promote plant growth [74]. The contents of total phenolics, flavonoids and anthocyanin were noticeably increased in both pea varieties by foliar application of AsA. Ascorbic acid application enhanced membrane stability, which in turn triggered plant defence by altering the activity of enzymes, total phenolic compounds, and proline content, which scavenged ROS and shielded the cells and strengthened the defense system.

Present study reported a considerable increase in both shoot and root Na^+^ while a dramatical decrease for both shoot and root K^+^ and Ca^2+^ under salt stress. The plants are unable to absorb K^+^, Ca^2+^ and other necessary nutrients and water because of the altered osmotic pressure in plant cells caused by higher Na^+^ levels in the soil [17]. Ascorbic acid treatment markedly improved K^+^ and Ca^2+^ uptake while lowering Na^+^ concentrations in both pea varieties under both saline and control environment. The signaling molecules potassium (K^+^) and calcium (Ca^2+^) are crucial for maintaining osmotic balance and promoting plant development [75]. Treatments with AsA (AsA) might keep cell’s homeostasis and K^+^ and Ca^2+^ ions stable during stress phases; as a resulst, it is a crucial metabolic regulator by encouraging the elimination of Na^+^ [76].

Ascorbic acid alleviates the effects of salinity through various mechanisms. Initially, AsA reduces oxidative damage brought on by salt stress by activating the antioxidant defense system and boosting the activity of enzymes including POD, CAT and SOD [77]. Furthermore, it enhances photosynthetic efficiency by safeguarding chloroplasts from oxidative stress and maximizing the absorption of CO_2_ [25, 78, 79]. Ascorbic acid also prevents ion imbalance and osmotic stress by modifying ion transporters, which lessens the hazardous accumulation of sodium ions (Na^+^) and increases the intake of essential nutrients like potassium (K^+^) and calcium (Ca^2+^) [80]. By encouraging the accumulation of compatible solutes like proline and soluble sugars, it maintains cellular turgor pressure and water uptake, facilitating osmotic adjustment and improving plant tolerance to salt stress [46, 65]. These mechanisms work together to increase plant growth, development and stress tolerance under saline environments.

In both varieties of pea, salt stress dramatically reduced yield attributes i.e., pod length, number of pods and 100 seed weight in current study. Plant development is noticeably stunted as a result of saline stress [81], because of the soil’s lower osmotic capacity that causes toxicity by modifying the actions of many enzymes involved in the metabolism of nucleic acids [82], affects protein metabolism [41], upsets hormonal balance and limits seed reserve utilization [83]. Foliar spray of AsA considerably improved yield attributes in both varieties of pea grown in both saline and control environment. The effect of AsA on yield may be connected to their important role in stress tolerance as it improves salt stress tolerance by decreasing oxidative stress, which stimulates plant growth, photosynthesis, nutrient absorption and eventually yield [66, 84].

Present study emphasized the negative impacts of increasing soil salinity, which inhibited pea growth and development by means of osmotic and ionic stress mechanisms. However, AsA application reduced these effects by improving morphological parameters, photosynthetic pigments and enzymatic antioxidant activities, thus improving plant resistance to salinity stress. Additionally, the application of AsA increases the production of secondary metabolites, osmolyte accumulation and endogenous antioxidant levels in peas, enhancing their yield attributes and stress tolerance. These findings contribute to a better understanding of pea responses to salt stress and emphasize ascorbic acid’s potential as a long-term strategy for increasing crop yield under stressful conditions.

## Conclusion

The current study was aimed to evaluate how two pea varieties cultivated in a salinized environment responded to exogenous foliar application of ascorbic acid on their morpho-physiological, biochemical and yield parameters. The findings of current study demonstrated that both 5- and 10-mM ascorbic acid levels were efficient and led to favourable results in comparison to the control treatment under both salinity and control conditions. However, it was shown that the foliar application of 10 mM ascorbic acid had more noticeable impact on the growth, photosynthetic pigments, enzymatic and non-enzymatic antioxidants, anthocyanin, flavonoids, and phenolic contents, resulting in an enhanced yield in salt-stressed pea plants. Comparing the two pea varieties, V2 (Sarsabz) outperformed V1 (Meteor) in terms of growth, yield, photosynthetic efficiency, soluble proteins, catalase activity, and shoot and root K^+^ as well as Ca^2+^ levels. In light of recent research, ascorbic acid at a concentration of 10 mM is suggested for promoting the growth and production of pea plants cultivated in salt-stressed soil. Future research might focus on conducting field trials to evaluate these findings on larger scales and in real-world scenarios. Furthermore, studying the long-term impacts of frequent ascorbic acid treatment, as well as its interactions with other management methods or stressors, could provide valuable insights into optimizing pea production in salt-affected areas.

## Data Availability

All data generated or analyzed during this study are included in this published article.
